# Tetrahydrofurandiols (THF-diols), Leukotoxindiols (LTX-diols), and Endocrine Disruption in Rats

**DOI:** 10.1289/ehp.9311

**Published:** 2007-01-29

**Authors:** Barry M. Markaverich, Mary Alejandro, Trellis Thompson, Shaila Mani, Andrea Reyna, Wendy Portillo, John Sharp, John Turk, Jan R. Crowley

**Affiliations:** 1 Department of Molecular and Cellular Biology and; 2 Center for Comparative Medicine, Baylor College of Medicine, Houston, Texas, USA; 3 Mass Spectrometry Facility, Department of Medicine, Washington University Medical School, School of Medicine, St. Louis, Missouri, USA

**Keywords:** corncob bedding, endocrine disruptors, LTX-diols, THF-diols

## Abstract

**Background:**

Ground corncob animal bedding and corn food products contain substances that disrupt endocrine function in rats. The disruptors were identified as isomeric mixtures of tetrahydrofurandiols (THF-diols; 9,12-oxy-10,13-dihydroxyoctadecanoic acid and 10,13-oxy-9,12-dihydroxyoctadecanoic acid) and leukotoxindiols (LTX-diols; 9,10-dihydroxy-12-octadecenoic acid and 12,13-dihydroxy-9-octadecenoic acid). The authentic compounds blocked sexual behavior in male rats and estrous cyclicity in female rats at oral doses of 2 ppm.

**Objectives:**

To define the lowest observed adverse effect level (LOAEL) for the THF-diols and LTX-diols in rats, we examined the nature of their interaction (additive or synergistic) and quantified the concentration of THF-diols in rat tissues.

**Methods:**

Adult male and female rats were provided drinking solutions containing various doses of THF-diols and/or LTX-diols, and we evaluated their effects on male sexual behavior and female estrous cyclicity. Tissues were collected for THF-diol determination by gas chromatography–mass spectrometry.

**Results:**

The LOAEL for THF-diols and LTX-diols for blocking estrous cyclicity was 0.5–1.0 ppm and 0.2–0.5 ppm, respectively. Higher concentrations (1–2 ppm) of THF-diols were required to block male sexual behavior. Combination studies with subthreshold doses of 0.05 ppm THF-diols plus 0.05 ppm LTX-diols revealed that their effects on estrous cyclicity were not synergistic. We were unable to detect THF-diols in tissues from rats treated with 10 ppm of the compounds, suggesting that metabolism may be involved.

**Discussion:**

THF-diols, LTX-diols, and/or their metabolites likely act additively to disrupt endocrine function in male and female rats at concentrations (0.5–1 ppm) that are 200-fold lower than those of classical phytoestrogen endocrine disruptors.

Reports from our laboratory and others have described adverse effects of corncob bedding on reproductive function in rats and mice (Markaverich et al. 2002b; Port and Kaltenbach 1969). For this reason, we recently identified one class of biological agents responsible for blocking male (Mani et al. 2005) and female sexual behavior and cyclicity in adult Holtzman rats (Markaverich et al. 2002a) as an isomeric mixture of 9,12-oxy-10,13-dihydroxyoctadecanoic acid and 10,13-oxy,9,12-dihydroxyoctadecanoic acid (tetrahydrofurandiols; THF-diols; Figure 1). The compounds were synthesized and shown to block male and female sexual behavior and cyclicity at an oral dose of 2 ppm when provided to rats over a 30-day period as a drinking solution. Although Halarnkar et al. (1989, 1992) demonstrated that high (millimolar) concentrations of THF-diols are toxic to insect cells, our studies suggest that at much lower concentrations, they are very active endocrine-disruptive agents in rats that can be purified on the basis of their mitogenic activities in human breast and prostate cancer model systems (Markaverich et al. 2002a). That a similar activity was also described in extracts of fresh corn or cob, and in corn tortillas purchased from a local supermarket further suggested that human exposure was possible (Markaverich et al. 2002a). Therefore, these compounds could represent a significant human health problem if their effects in rats are mirrored in humans (Markaverich et al. 2002b).

Further characterization of corncob extracts by HPLC led to the isolation, purification, and identification of a second HPLC-peak component with mitogenic activity from ground corncob bedding that also blocked cyclicity in female rats. We identified this activity as 9,10-dihydroxy-12-octadecenoic acid (LTX-diol), a well-known leukotoxin-diol (Markaverich et al. 2005). Like the THF-diols, synthetic preparations of LTX-diols blocked cyclicity in female rats at oral doses of 2 ppm; however, these preparations failed to block male sexual behavior. Thus, sex differences may exist in the response profiles of the THF-diols and LTX-diols in the rat.

These earlier studies with synthetic THF-diols or LTX-diols only assessed the effects of a single, higher dose (2 ppm) of these compounds on reproductive function in male (sexual behavior; THF-diols) and female (lordosis and ovarian cyclicity; THF-diols and LTX-diols) rats to confirm the identity of these endocrine-disruptive substances; these higher doses completely ablated the response in these various systems (Markaverich et al. 2002a, 2005). Therefore, we were unable to establish a lowest observed activity level (LOAEL) of the THF-diols and/or LTX-diols responsible for the disruption of endocrine function in these experimental systems. The studies we describe in this article were performed to more precisely define the individual and relative endocrine-disruptive activities of the THF-diols and LTX-diols in female and male rats. We also attempted to determine the levels of the THF-diols present in tissues responsible for their biological activity, and we evaluated the nature of the interaction (additive or synergistic) of THF-diols and LTX-diols when administered to female rats in a combined fashion, as would be the case during exposure to corncob bedding material or other corn preparations.

## Materials and Methods

### Reagents and solvents

Linoleic acid, ^13^C_18_-linoleic acid, *m*-chloroperoxybenzoic acid (mCPBA), and tetrahydrofuran (THF) were purchased from Sigma Chemical Company (St. Louis, MO). We purchased Sep-Pak C_18_ cartridges (3 cc) from Waters Corporation (Milford, MA) and C_18_ mini-columns from Varian (Walnut Creek, CA). All solvents were HPLC grade from Burdick and Jackson (Muskegon, MI). *N*,*O*,-bis(trimethylsilyl)trifluoroacetamide with 10% trimethylchlorosilane (N,O,-bis(trimethylsilyl)trifluoroacetamide; BSTFA) was obtained from Pierce Chemical (Rockford, IL).

### Animals and treatment

For the estrous cycle studies in female rats, we used adult Holtzman Sprague-Dawley male and female rats (Holtzman Laboratories, Madison, WI). The animals were housed in suspended stainless steel wire cages and were maintained in compliance with federal guidelines for animal care (Human Health Extension Act of 1985; National Institutes of Health 2002). All procedures received appropriate institutional animal care and use committee approval, and animals were treated humanely with regard for alleviation of suffering. The room was maintained under climate-controlled conditions with a 12-hr:12-hr light/dark cycle (lights on at 0600 hours). Food (Harlan Teklad Global Diet no. 2014 containing no alfalfa, soybean, or phytoestrogens; Harlan Teklad, Madison, WI) and water were provided *ad libitum*. Reversed lighting conditions were used for sexual behavior studies. Administration of compounds to male and female rats and assessment of effects on reproductive function varied by experiment.

### Evaluation of the effects of THF-diol and LTX-diol on the estrous cycle

The methods and experimental protocols used to assess the effects of THF-diol and LTX-diol on the estrous cycle in female rats were as previously described (Markaverich et al. 2002a, 2002b, 2005) with slight modifications, as indicated in the text and figures. For each of the studies assessing THF-diol and/or LTX-diol effects on the estrous cycle, daily vaginal smears were collected from groups of 8–9 adult female cycling rats given tap water–2% Tween-80 vehicle (vehicle controls) or 1:1 mixtures of THF-diols or LTX-diols dissolved in vehicle at 0.2, 0.5, or 1.0 ppm. This vehicle has no significant effects on estrous cyclicity, body weights, or fluid consumption relative to tap water controls (Markaverich et al. 2002a, 2002b, 2005). These doses were chosen on the basis of earlier studies demonstrating that oral administration of 2-ppm concentrations of THF-diols or LTX-diols completely blocked male and female sexual behavior and ovarian cyclicity in Holtzman female rats of this age.

**Figure 2:**
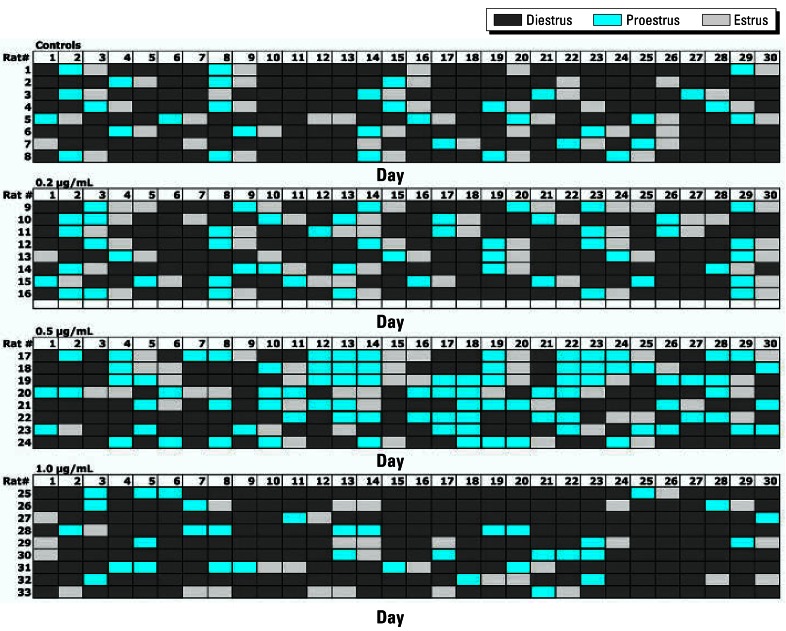
Effects of THF-diols on the estrous cycle of adult female Holtzman rats treated with vehicle (controls) or various doses (0.2, 0.5, or 1 ppm) of approximately 1:1 mixture of THF-diol isomers. See “Materials and Methods” for details. Of the vehicle controls, 100% (8/8) displayed normal (5–7 day) cycles, whereas cyclicity was suppressed in the 0.2-ppm (75% normal cycles; 6/8 rats), 0.5-ppm (87.5% normal cycles; 7/8), and 1.0-ppm (22% normal cycles; 2/9 rats) treatment groups. Four rats in the 1.0-ppm group displayed a single estrus smear during the 30-day period.

**Figure 3:**
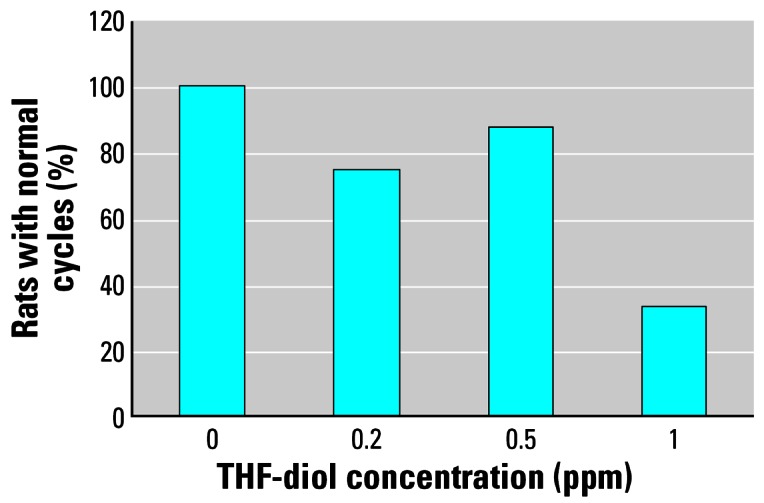
Number of female rats displaying normal (5–7 day) estrous cycles after treatment with vehicle or 0.2, 0.5, or 1 ppm THF-diol. See “Materials and Methods” for details.

**Figure 4:**
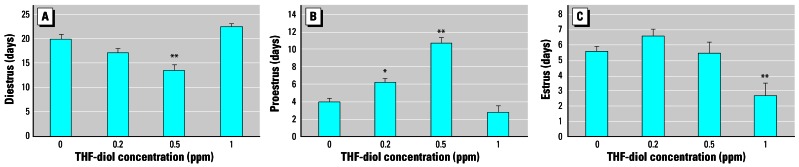
Effects of THF-diols on the duration (number of days) of diestrus, proestrus, and estrus during a 30-day period in female rats treated with vehicle or 0.2, 0.5, or 1 ppm THF-diol. *(A*) diestrus. *(B*) Proestrus. *(C*) Estrus. Results are expressed as mean ± SE; data were analyzed statistically as described in “Materials and Methods.” *p < 0.01, and **p < 0.001, compared with all other groups.

**Figure 5:**
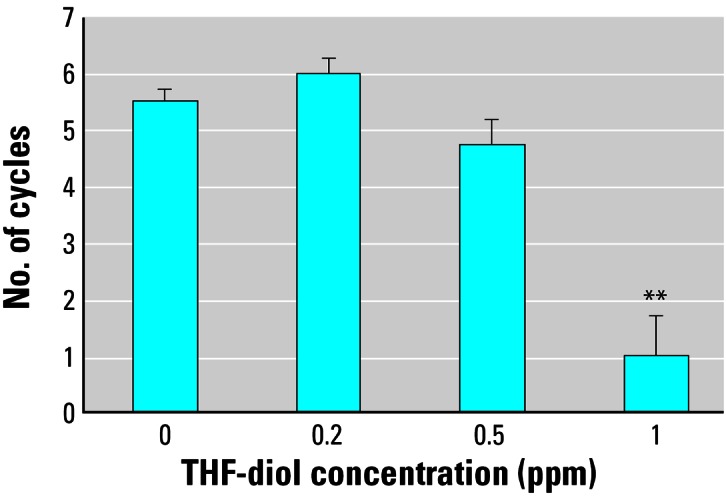
Effects of THF-diol on the number of normal estrous cycles (5–7 days) in adult female Holtzman rats during the 30-day period for the groups described in Figure 2. Results are expressed as mean ± SE; data were analyzed statistically as described in “Materials and Methods.” **p < 0.001 compared with vehicle control.

**Figure 6:**
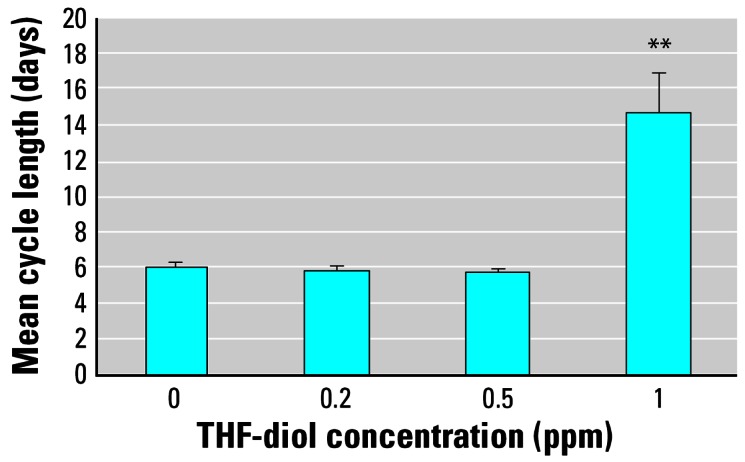
Effects of THF-diol on estrous cycle length in adult Holtzman rats as described in Figure 2. Results are expressed as mean ± SE; data were analyzed statistically as described in “Materials and Methods.” **p < 0.001 compared with all other groups.

Preliminary dose–response studies with the LTX-diols (not shown) served as the basis for the design of the dose–response studies presented in Table 1. In addition, the preliminary studies facilitated the estimation of sub-LOAEL doses of the LTX-diols to be used for combination studies with THF-diols to explore the nature of the interaction (additive or synergistic) of the two classes of compounds. The sub-LOAEL doses of THF-diols were also determined in the preliminary studies (Figures 2–6). Consequently, we were able to design experiments (Table 1) with five groups of rats (*n* = 8/group). The dose–response component of the study included four groups: vehicle controls, and 0.2, 0.5, and 1.0 ppm LTX-diols. An additional group received a combination of 0.05 ppm THF-diols plus 0.05 ppm LTX-diols to assess the effects of sub-LOAEL doses of the compounds on cyclicity relative to the vehicle control. Thus, the dose–response study and drug combination studies were run simultaneously, using a single group of controls to minimize costs and labor. This study allowed us to estimate the LOAEL for the LTX-diols and to explore the nature of the interaction of the THF-diols and LTX-diols in this model system.

### Collection of vaginal smears

Daily vaginal smears were collected throughout the 30-day period as previously described (Markaverich et al. 2002a, 2002b, 2005). Cytology was evaluated and stage of the cycle determined as described by Everett (1989). Diestrus smears contained numerous leukocytes, Schorr cells, increasing basophilic epithelial cells, and mucous. Proestrus smears were deficient in leukocytes and contained few to many clustered basophilic epithelial cells, few nucleated epithelial cells, and increasing bacteria. Estrus smears were deficient in leukocytes and contained decreasing bacteria and sheets or clumps of cornified epithelial cells. Data were analyzed by methods recently described by Biegel et al. (1998) to evaluate THF-diol and/or LTX-diol effects on the estrous cycle in rats. The number of days in each stage of the cycle, cycle length, and the numbers of normally cycling females in each group were determined. A normal cycle in these Holtzman rats housed in wire cages was 5–7 days, including 1–2 days of estrus. A complete estrous cycle was defined as the day after estrus to the day after the subsequent estrus.

### Assessment of THF-diol effects on male sexual behavior

For behavioral studies, we used adult Harlan Sprague-Dawley rats (males weighing 300–320 g and ovariectomized females weighing 160–180 g) purchased from Harlan (Houston, TX). The animals were housed in suspended wire cages on a 12 hr:12 hr reversed light cycle with lights off at 12:00 hours. Food and water were provided *ad libitum*. THF-diol isomers (Markaverich et al. 2002a) were dissolved in tap water containing 2% Tween-80 and administered as drinking solution. Experimental groups received 2% Tween-80 (vehicle) or 0.2 or 1.0 ppm THF-diol isomers in vehicle for 2 weeks. There were no significant effects of this vehicle on male or female reproductive function compared with standard drinking water (Mani et al. 2005; Markaverich et al. 2002a). Fluid consumption and body weights were monitored daily to assess treatment effects on these parameters. We observed no overt signs of toxicity (weight loss, decreased feed or water consumption), hair loss, or incontinence. LTX-diols had previously failed to affect male sexual behavior (Markaverich et al. 2005); therefore, they were not studied in further detail here.

### Behavioral testing

Behavioral tests were conducted in a 50 × 45 × 25 cm Plexiglas arena. Stimulus females were rendered sexually receptive with subcutaneous (sc) injections of estradiol benzoate (2 μg) in sesame oil followed 48-hr later with 100 μg progesterone (sc). Behavioral tests were conducted 4–6 hr after progesterone treatment of the stimulus females. Male rats, which had consistently ejaculated on three previous screening tests, were selected for further experiments. Each test lasted for 30 min after the introduction of the stimulus female into the arena. We recorded the number of attempted mounts (M), mount frequency (MF; number of mounts with pelvic thrusts), mount latency (ML; latency from the introduction of the female to the first mount), intromission latency (IL; latency from the introduction of the female to the first intromission), ejaculation latency (EL; latency from the first intromission to the first ejaculation), intromission frequency (IF; number of mounts with vaginal insertion to an ejaculation), ejaculation frequency (EF; number of ejaculations per test), post ejaculatory interval (PIL; latency from the first ejaculation to the first intromission of second ejaculatory series); number of thrusts per intromission (T/I), and grooming frequency (GF; number of grooming responses after intromissions). Behaviorally, intromissions were distinguished from mounts without intromissions by the occurrence of deep thrust followed by rapid, springing dismount. Ejaculation patterns were characterized by longer, deep thrusts, slow dismounts and a prolonged period of rest after dismount. Observers were blind to the treatments administered.

### Synthesis of THF-diols and LTX-diols for oral dosing studies in rats

Approximately 1:1 isomeric mixtures of 9,12-oxy-10,13-dihydroxyoctadecanoic acid and 10,13-oxy-9,12-dihydroxyoctadecanoic acid (THF-diols) or 9, 10-dihydroxy-12-octadecenoic acid and 12,13-dihydroxy-9-octadecenoic acid (LTX-diols) for the oral dosing studies were synthesized from linoleic acid by previously reported methods (Markaverich et al. 2005). The THF-diol and LTX-diol isomers were purified to homogeneity on C_18_-reversed phase columns and structurally confirmed by gas chromatography–mass spectrometry (GC-MS) analyses (Markaverich et al. 2005).

### Synthesis of ^13^C_18_-THF-diols as an internal standard for GC-MS analyses

The epoxy acids were synthesized from ^13^C_18_-linoleic acid by incubating 10 mg ^13^C_18_-linoleic acid in the presence of mCPBA (70 mg) at 22°C for 5 hr. The reaction product was washed sequentially with 1-mL volumes of 25 mM ammonium carbonate, saturated saline solution, and H_2_O and the washed product was dried under N_2_. The waxy white solid was redissolved in THF:H_2_O:5% perchloric acid (5:1:1) and stirred at ambient temperature for 1 hr. The products were extracted into ethyl acetate and evaporated to dryness in a speed vacuum. The reaction products, including the ^13^C_18_-9,12-oxy-10,13-dihydroxyoctadecanoic acid and ^13^C_18_-10,13-oxy-9,12-dihydroxyoctadecanoic acids, were redissolved in ~ 300 μL 30% acetonitrile (CH_3_CN)–0.5% acetic acid and loaded onto a 10-g C_18_ column preequilibrated with 2 mL CH_3_CN and 4 mL 0.5% acetic acid. The column was washed with 30% CH_3_CN in 0.5% acetic acid to elute residual by-products of the mCPBA not removed by extraction. The ^13^C_18_-THF-diols were eluted from the column with 60% CH_3_CN in 0.5% acetic acid, and their structure and purity was confirmed by GC-MS (Markaverich et al. 2002a, 2005).

### GC-MS analysis of tissue extracts for THF-diols

Tissues including the hypothalamus, pituitary, uterus, ovary, adrenal gland, kidney, liver, and skeletal muscle (gastrocnemius) were collected from female rats (*n* = 8–10/group) treated for 30 days with vehicle or 2 ppm or 10 ppm THF-diols administered as drinking solutions. The animals were sacrificed and the various tissues were collected, rinsed in chilled 0.9% saline, stripped of extraneous material, blotted, weighed, and stored at −20°C prior to analysis for THF-diols by GC-MS. Approximately 30 mg frozen tissue from vehicle controls or rats treated with THF-diol (2 ppm or 10 ppm for 30 days) were homogenized in a hand-held ground glass homogenizer in 0.5 mL 50 mM disodium phosphate. The tissue samples were spiked with 10 μL ^13^C_18_-THF-diols (720 pmol) as an internal standard, incubated at 22°C for 2 hr, and stored at −20°C for 16–18 hr. The frozen homogenates were thawed, the pH adjusted to 1 with 20 μL HCl (12 N), and the lipids were extracted for 10 min with chloroform: methanol (2:1). The sample extracts were centrifuged for 5 min at 1,000 × *g*, and the chloroform layer was taken to dryness under vacuum. The tissue extracts were derivatized with 100 μL BSTFA/CH_3_CN (1:3) at 70°C for 15 min and 2 μL of each sample was analyzed on a Varian 3400 (Varian Inc., Palo Alto, CA) gas chromatograph interfaced to a Finnigan SSQ 7000 mass spectrometer (Thermon Scientific, Waltham, MA). We used a DB-1 GC column (15 m, 0.2 mm i.d., 0.33-μm film coating, P.J. Cobert, St. Louis, MO). We used a linear temperature program: The initial temperature of 120°C was held for 1 min and increased to 270°C at 15°/min; the temperature was then held at 270°C for 1 min. For electron ionization the source temperature, electron energy, and emission current were 200°C, 100eV, and 300 μA, respectively. The injector and transfer line temperatures were 250°C.

### Statistical analyses

For the cycling studies in female rats, data were analyzed statistically by analysis of variance (ANOVA) and the appropriate multiple range test on treatment means (Instat; GraphPad Software, San Diego, CA). The data recorded from the behavioral tests were compared using Kruskal-Wallis ANOVA followed by Dunn’s method for post hoc comparison.

## Results

### Estimation of THF-diol delivery to rats as a drinking solution

Oral delivery of THF-diols as a drinking solution (2.0 ppm) completely blocked sexual behavior in male and female rats and estrus cyclicity in the rat (Markaverich et al. 2002a). A major goal of the present studies was to more precisely define the biological activity of THF-diols and determine the LOAEL. For this reason, adult female rats (*n* = 8–9/group) were treated for 30 days with drinking solutions containing 0.2, 0.5, or 1.0 ppm THF-diols in 2% Tween 80 vehicle. Fluid consumption and body weights were monitored every 6–8 days throughout the study. The dose of THF-diols delivered to the animals was calculated using these parameters. The data showed that the 0.2-, 0.5-, and 1.0-ppm THF-diol drinking solutions delivered approximately 0.04 ± 0.003, 0.1 ± 0.006, and 0.175 ± 0.016 mg/kg/rat/day, respectively, to the female rats. This dose–response curve was linear, indicating very good delivery. During the 2-week breeding study with male rats, the 0.2- and 1.0-ppm doses of THF-diols delivered approximately 0.025 ± .002 and 0.130 ± 0.011 mg/kg/rat/day, respectively. The difference in delivery between male and female rats reflects differences in body weights and fluid consumption by the two sexes. We observed no significant effects on the body weights of these male and female animals treated with THF-diols (not shown), confirming that toxicity was not a problem.

### THF-diol effects on the estrous cycle of adult female rats

We performed the dose–response studies to more accurately assess the LOAEL of the THF-diols and further define their toxicity. Figure 2 is a schematic of the cyclicity data collected from the vehicle controls and the 0.2-, 0.5-, and 1.0-ppm THF-diol treatment groups administered the compounds for 30 days as drinking solutions. We used these data to generate Figures 3–6. Figure 3 shows that 100% (8/8) of the vehicle-treated controls displayed normal 5- to 7-day cycles. This cycle length is consistent with that of rats housed in our animal facility in suspended wire cages. Treatment with 0.2 ppm (6/8 rats cycling normally) or 0.5 ppm (7/8 cycling normally) THF-diols slightly reduced cyclicity, whereas a substantial reduction (78%) in the numbers of rats (2/9) displaying normal 5- to 7-day cycles was observed following treatment with 1.0 ppm THF-diols (Figure 3).

We also assessed treatment effects on specific phases of the estrous cycle. The data in Figure 4A suggest that treatment with 0.5 ppm THF-diols significantly decreased the numbers of days of diestrus during the 30-day treatment period. Similarly, both the 0.2 and 0.5 ppm doses increased the numbers of days the rats were in proestrus (Figure 4B), even though these treatment regimes failed to affect the number of days the rats were in estrus (Figure 4C), the number of cycles observed in the 30-day period (Figure 5), or the length of the estrous cycle (Figure 6). Consequently, the lower doses may have slightly “pushed” the rats more toward proestrus, reducing the duration of diestrus. Conversely, treatment with 1.0 ppm THF-diol failed to significantly affect the length of diestrus (Figure 4A) or proestrus (Figure 4B); however, this dose significantly decreased (*p* < 0.001) the number of days the rats were in estrus (Figure 4C) and the number of estrous cycles (Figure 5; *p* < 0.001) and significantly increased (*p* < 0.001) cycle length (Figure 6). The 1.0-ppm dose of THF-diols blocked estrus in 78% (7/9) of the animals relative to the controls or the lower dose treatment groups, and only 2 animals receiving 1.0 ppm THF-diols displayed normal 5- to 7-day cycles. Cycle length in the 1.0-ppm THF-diol group (Figure 6; 14.25 days) was significantly longer (*p* < 0.001) than that observed in the controls (5–7 day range; Figure 6) or lower dose groups. These data indicate that 0.5–1.0 ppm approximates the true LOAEL of THF-diols in this experimental system when given as a drinking solution. This level is substantially lower than that obtained with classical endocrine disruptors including phytoestrogens and micotoxins that typically require much higher oral concentrations (approaching 500 ppm) (Markaverich et al. 1995).

### Effects of THF-diol on male sexual behavior

**Figure 7:**
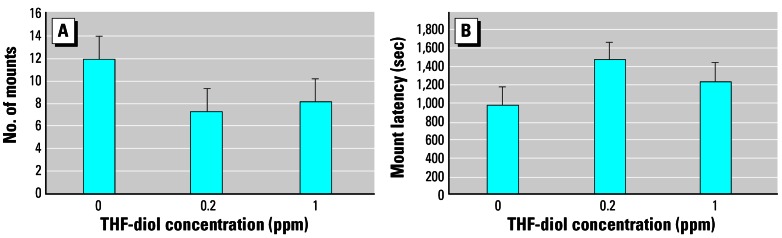
Effects of THF-diol on mounting frequency (*A*) and mounting latency (*B*) in male rats given vehicle (controls), or 0.2 or 1.0 ppm THF-diols for 14 days. Results are expressed as mean ± SE for triplicate determinations from 6 rats for each dose level; data were analyzed statistically as described in “Materials and Methods.” No significant treatment effects were observed, although the decrease in mounting frequency approached significance in both THF-diol treatment groups.

An earlier study from our laboratory (Mani et al. 2005) demonstrated that an oral dose of 2 ppm THF-diols completely blocked a number of male sexual behavior responses including mounting frequency and mounting latency. In the present study we reevaluated these earlier studies using lower oral doses of THF-diols, and observed no significant treatment effects on mounting frequency (Figure 7A) or mounting latency (Figure 7B) with either the 0.2- or 1.0-ppm THF-diol dose. Nevertheless, both doses slightly decreased mounting frequency and increased mounting latency to levels just approaching significance, suggesting that this dose range was marginal for response. Nil responses (data not shown) were observed for the number of attempted mounts, intromission latency, ejaculation latency, intromission frequency, ejaculation frequency, postejaculatory interval, number of thrusts per intromission, and grooming frequency, as previously described (Mani et al. 2005; Markaverich et al. 2002a, 2002b). Therefore, the LOAEL for the THF-diols in male rats is likely to be equivalent to the 2-ppm concentration previously reported to block these responses (Mani et al. 2005), because the 0.2–1.0 ppm doses used in the present studies very marginally modulated male sexual behavior.

### Determination of tissue levels of THF-diols

A major goal of this study was to determine the tissue levels of THF-diols associated with a disruption of the estrous cycle. For this reason, we analyzed a number of tissues from female rats treated with THF-diols: hypothalamus, pituitary, uterus, adrenal gland, ovary, kidney, liver, and skeletal muscle. Initially, tissues were collected from adult female rats treated with 2 ppm THF-diols for 30 days, because this dose completely blocked estrous cyclicity (Markaverich et al. 2002a). However, we were unable to detect these compounds in tissue extracts by GC-MS analyses. Consequently, a second study was performed and tissues were collected from female rats treated for 30 days with 10 ppm THF-diols, which are 10-to 50-fold higher than those used in the previous study. Nevertheless, we were unable to detect THF-diols in these tissue extracts, even though detection of the ^13^C_18_-THF-diol internal standards added to the samples before homogenization and extraction was exceptional and in the range of 5 pmol (1.65 ng). At this THF-diol/tissue mass ratio, this is approximately 0.165 ppm.

### Dose-dependent LTX-diol effects on the estrous cycle

**Figure 8:**
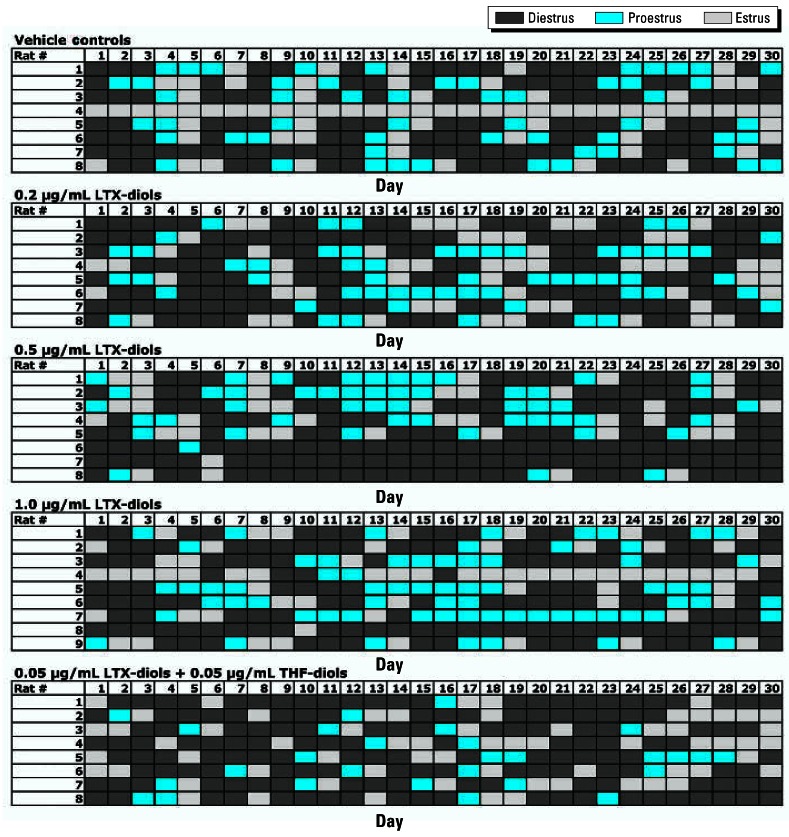
Effects of THF-diols on the estrous cycle of adult female Holtzman rats treated with vehicle (controls) or with LTX-diols (0.2, 0.5, or 1.0 ppm; dose–response study) or LTX-diols plus THF-diols (0.05 ppm each; combination study) as described in “Materials and Methods.” The data for the dose–response study are also summarized in Table 1. Data for the combination study are summarized in Figure 9.

Drinking solutions containing 2 ppm LTX-diols completely blocked cyclicity in female rats, even though they failed to affect male sexual behavior (Mani et al. 2005; Markaverich et al. 2005). Because the LOAEL of LTX-diols required to block estrus is unknown, we evaluated the effects of a range of LTX-diol doses on cyclicity in female rats to estimate this parameter. A mixture of LTX-diol and isoleukotoxin diol (iso-LTX-diol) was used because the chemical synthesis protocol routinely generates nearly equimolar mixtures of both isomers (LTX-diol + iso-LTX-diol) that have indistinguishable biological activities in our experimental systems *in vitro* (Markaverich et al. 2005). The results of the LTX-diol dose–response studies are shown in Figure 8 and summarized in Table 1. This analysis was equivalent to that shown for the THF-diols (Figures 2–6). The data in Table 1 show that at doses of 0.2–0.5 ppm, LTX-diol treatment significantly decreased (*p* < 0.01) the number of 5- to 7-day estrous cycles in the 30-day period relative to controls, and also decreased the numbers of rats with normal estrous cycles by nearly 60%. Therefore, the LOAEL of the LTX-diols, when given as an oral solution, is approximately 0.2–0.5 ppm. This is close to that observed for the THF-diols in the studies described above.

### Effects of combination THF-diol and LTX-diol treatment on the estrous cycle

**Figure 9:**
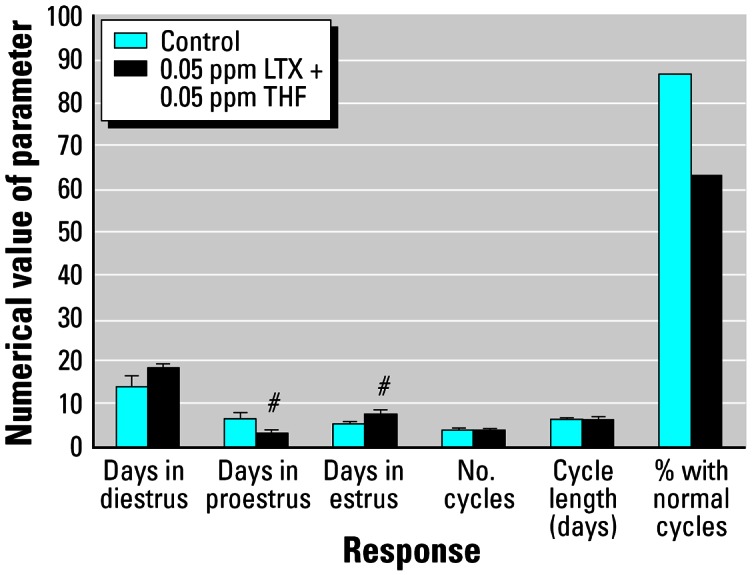
Effects vehicle (control) or THF-diols plus LTX-diols (0.05 ppm each; combination study) on the estrous cycle of adult female Holtzman rats (*n* = 8/group) evaluated as described in “Materials and Methods” and shown in in Figure 8. The combined dose level (0.1 ppm) of THF-diols and LTX-diols was equivalent to the LOAEL of the compounds (Figures 2–6 and 8), which significantly blocked estrous cyclicity. The minimal response to combined THF-diol plus LTX-diol treatment rules out synergistic interactions of these compounds in this experimental system, and suggests that their effects are additive. ^#^*p* < 0.05.

Both THF-diols and LTX-diols have been identified as endocrine-disruptive components in ground corncob bedding. However, we were unable to define the nature of their combined effects on reproductive function in these crude preparations. The availability of significant quantities of the pure isomeric mixtures and the completion of the dose–response studies allowed us to evaluate their combined effects on the estrous cycle. The data in Figures 8 and 9 illustrate the combined effects of 0.05 ppm THF-diols plus 0.05 ppm LTX-diols on the estrous cycle in Holtzman rats relative to vehicle controls. These doses were intentionally chosen to be subthreshold such that, if a synergistic interaction occurred, the observed response would be much greater than that observed at 0.5–1.0 ppm, the LOAELs determined for these compounds. Although the combined dose (0.1 ppm total) of THF-diols plus LTX-diols slightly affected the durations of proestrus and estrus, and reduced the numbers of rats with normal cycles by about 20% relative to controls, this was likely an additive, but not synergistic, response. A synergistic response would have been expected to profoundly affect estrous cyclicity, as was the case for 2-ppm doses of THF-diols or LTX-diols (Markaverich et al. 2002b, 2005); this type of response was not observed. These data argue for a common mechanism of action for the THF-diols and LTX-diols and effectively rule out synergistic effects. Thus, on the basis of the dose–response studies and the combination studies we performed (Figures 2–6, 8, and 9), it appears that the toxicologic activities of THF-diols and LTX-diols in ground corncob bedding materials and food products will likely be directly proportional to the sum of their individual concentrations and synergistic interactions are unlikely.

## Discussion

Our laboratory described two classes of endocrine disruptors in ground corncob animal bedding with mitogenic activities in breast and prostate cancer cells *in vitro* and *in vivo* (Markaverich et al. 2002b). These two classes of disruptors were recently purified to homogeneity and identified as isomeric mixtures of THF-diols (Markaverich et al. 2002a) or LTX-diols (Markaverich et al. 2005). THF-diols and LTX-diols block female sexual behavior and cyclicity and stimulate breast cancer cell proliferation. Interestingly, the THF-diols, but not the LTX-diols, block male sexual behavior (Mani et al. 2005; Markaverich et al. 2005). The mechanism of action of these compounds remains to be resolved, although we suspect their effects on estrous cyclicity and sexual behavior in male and female rats are mediated via the modulation of nitric oxide–dependent pathways controlling gonadotrophin-releasing hormone release (Hull et al. 1994; Ishizaki et al. 1995a, 1995b; Moses and Hull 1999). The present studies support a common mechanism of action.

THF-diols and LTX-diols possess antagonistic activity in the reproductive tract (uterus and vagina) even though the compounds do not bind to the estrogen receptor (Markaverich et al. 2002a, 2002b, 2005). Housing adult female rats on ground corncob bedding causes significant reductions in uterine weight and DNA content relative to controls (Markaverich et al. 2002b). Drinking solutions containing 2 ppm concentrations of THF-diols or LTX-diols completely (100%) blocked estrus in rats, such that no definitive measure of LOAEL or mechanism(s) of action or interaction (additive versus synergistic) of the THF-diols or LTX-diols could be obtained. Because LTX-diols and THF-diols are components of corncob bedding and corn products, it is critical that their individual and combined activities and toxicologic properties be determined. The limited availability of milligram quantities of the THF-diols or LTX-diols prevented us from evaluating their biological activities *in vivo* over a range of concentrations for sustained periods of time. We recently developed methods for synthesizing milligram quantities of highly purified THF-diols or LTX-diols (Mani et al. 2005) that provided sufficient material for these experiments.

In the present studies, we found dose-dependent effects of THF-diols on the estrous cycle of Holtzman Sprague-Dawley rats housed in stainless steel wire cages. Although the 0.2- to 0.5-ppm doses have subtle effects on the number of days of diestrus or proestrus (Figure 4A, 4B), these levels failed to significantly alter the number of days in estrus (Figure 4C), the number of estrous cycles (Figure 5), or the duration of the estrous cycle (Figure 6). The lower doses did reduce the days in diestrus and increase the days in proestrus, suggesting subtle effects on ovarian hormone production. Alternatively, the 1.0-ppm THF-diol dose failed to modify the days in diestrus or proestrus, but clearly blocked the estrous cycle in these animals (Figures 2, 4C, and 5) and increased the duration of time between cycles from the 5–7 days for vehicle controls or low-dose animals to 14.25 days in the high-dose group (with the exception of two animals with 5- to 7-day cycles in the high-dose group; Figure 2). Thus, the THF-diols reduced the duration of “estrogen-responsive phases” of the estrous cycle. We suspect that this was caused by a decrease in circulating estrogens. We did not monitor blood levels of ovarian steroids throughout the course of the studies because the interpretation of this type of data from nonsynchronized groups consisting of eight to nine rats would have been extremely difficult, particularly if the effects on circulating steroids were subtle. More precise molecular studies are in progress to define the mechanism of action of these compounds.

A major goal of the present studies was to define the LOAEL for these compounds, and this goal was accomplished. THF-diols at 1 ppm blocked cyclicity in approximately 78% (7/9) of the animals. Lower doses (0.2–0.5 ppm) had little measurable effect on cyclicity (Figure 3). Thus, the LOAEL of the THF-diols, when supplied as a drinking solution to adult Holtzman female rats, is approximately 0.5–1 ppm. This level is substantially lower than that observed (500 ppm) for classical phytoestrogens including coumestrol to modulate biological response in adult female rats (Markaverich et al. 1995). Thus, the THF-diols have substantial biological activity. In addition, the level of THF-diols [> 1 ppm; Figure 7A, 7B (Mani et al. 2005)] required to block biological response in male rats appears to be higher than that required to affect the estrous cycle in females (0.5–1.0 ppm). This could simply reflect differences in body weights, fluid consumption, and/or metabolic rates between the two sexes, or a difference in the duration of treatment. Metabolism of the compounds may also be involved in this differential response profile. It is clear that THF-diols are capable of disrupting endocrine function in male and female rats at very low oral concentrations (0.5–2.0 ppm), demonstrating that these are very active endocrine-disrupting substances.

The dose–response studies with the LTX-diols in female rats also indicate that the LOAEL of these compounds is in the 0.2–0.5-ppm dose range. These concentrations had subtle, but statistically significant, effects on the estrous cycle in rats (Table 1). Furthermore, the low-dose combination studies with the THF-diols plus LTX-diols revealed that synergistic interactions between these compounds are unlikely (Figure 9). The combined low doses minimally affected estrous cycle parameters. If the response were synergistic, the combined effects of the lower dose levels of THF-diols plus LTX-diols (0.1 ppm combined) would have far exceeded the response to 0.2–1.0 ppm of either of these compounds alone (Figures 2–6 and 8). This was not the case. Therefore, their combined response appears to be additive. This is consistent with the fact that both classes of compounds are chemically related and might be expected to have a common mechanism of action (Moghaddam et al. 1996, 1997).

THF-diols are likely to be derived from LTX-diols (Moghaddam et al. 1996, 1997), and for this reason we focused our initial studies on attempts to determine tissues levels of THF-diols in rats treated with these compounds. Assuming that THF-diols are not metabolized, conjugated, or rapidly excreted in rats, key target tissues should contain intact THF-diols. If 0.1% of the administered dose of the compounds was absorbed following oral consumption, rats treated with 0.2–1 ppm THF-diols should have absorbed 40–200 ng/day from drinking solutions. Similarly, rats drinking the 10-ppm THF-diol solution should have absorbed approximately 2 μg THF-diols per day. Even when exposed to these high concentrations (10 ppm), we were unable to quantify the THF-diols in a variety of tissues using GC-MS techniques capable of detecting < 2 ng of these compounds. It is unlikely that this was due to the inefficiency of our extraction procedures, because the tissues were spiked with ^13^C_18_-THF-diol internal standards before homogenization, and the recovery of ^13^C_18_-THF-diols from the tissues was > 70%. These findings suggest that if intact THF-diols accumulate in tissues, quantities < 2 ng/30 mg tissue were responsible for the biological activity. It is unlikely that the THF-diols in our studies were not detected because they were conjugated (glucuronides or sulfonates). Although diurnal fluctuations in linoleate-and arachidonate-derived metabolites, including LTX-diols and THF-diol–related compounds, are observed in human subjects, rodents apparently excrete the parent lipids (Newman et al. 2002). Perhaps the metabolism of THF-diols and/or LTX-diols is involved in the mechanism of action of these compounds, and studies are under way in our laboratories to evaluate this possibility. This would certainly be consistent with the observations that the THF-diols are capable of blocking female (Mani et al. 2005), but not male (Figure 7) sexual behavior. Perhaps male rats are incapable of generating an “active” THF-diol metabolite(s).

Regardless of the metabolic fate or mechanism of action of the THF-diols or LTX-diols, it is clear that exposure of rats to these compounds via the bedding material, ground corncob extracts, or oral administration of highly purified synthetic preparations disrupts male and female sexual behavior and cyclicity in the 0.5- to 1-ppm concentration range. We have not yet determined the concentration of the THF-diols or LTX-diols present in ground corncob bedding material or in human foods such as corn tortillas or fresh corn (Markaverich et al. 2002b). However, the present studies suggests that the compounds are capable of blocking the estrous cycle and reproductive function in rats at very low concentrations. Similar studies with THF-diols or LTX-diols in other mammalian species will provide important information regarding their ultimate impact on endocrine function in experimental animals and humans.

## Figures and Tables

**Figure 1:**
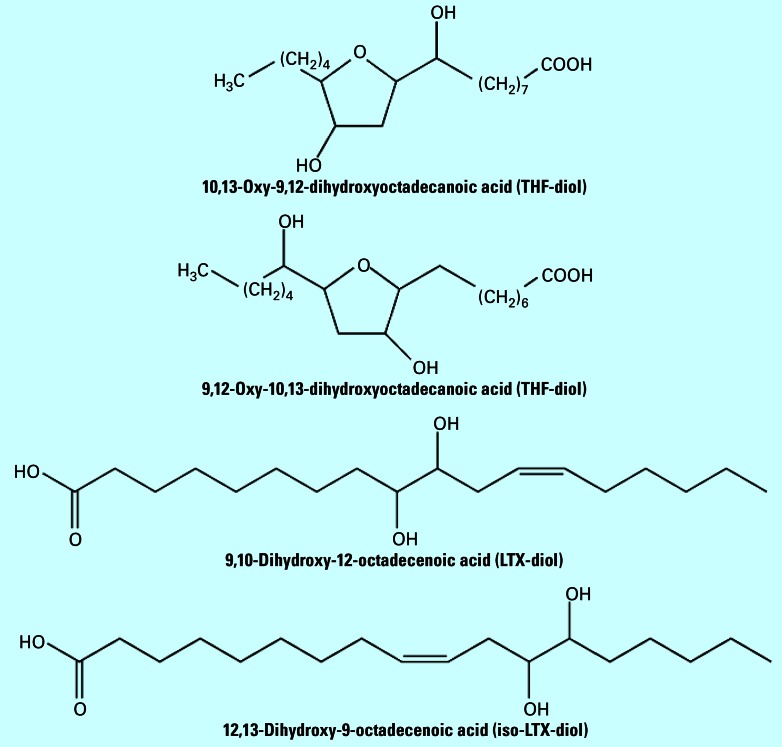
Structures of THF-diols and LTX-diols.

**Table 1:** Effects of LTX-diols on the rat estrous cycle.

LTX-diol concentration (ppm)	Daily dose delivered (mg/kg/day)	Diestrus (days)	Proestrus (days)	Estrus (days)	Mean cycle length (days)	Rats with normal cycles (%)	No. normal cycles in 30 days
0	0 ± 0	14.25 ± 2.32	7 ± 1.15	5.6 ± 0.5	6.65 ± 0.41	86.0	4.17 ± 0.17
0.2	0.29 ± 0.0005	15.8 ± 1.32	7.38 ± 1.36	7.6 ± 0.32	8.2 ± 1.25	63.0	4.0 ± 0.32
0.5	0.056 ± 0.0026	18.5 ± 2.6	6.4 ± 1.5	5.0 ± 1.16	5.17 ± 1.18	50.0	1.625 ± 0.5*
1	0.096 ± 0.0077	15.6 ± 2.31	6.67 ± 1.6	5.7 ± 0.73	5.53 ± 1.08	33.0	1.89 ± 0.56*

* Significantly different from control (0 μg/mL), *p* < 0.01.
